# Supplementary material for: The Construction and Exploration of a Comprehensive MicroRNA Centered Regulatory Network in Foxtail Millet (Setaria italica L.)
Source: Front Plant Sci. 2022 May 6;13:848474. doi: 10.3389/fpls.2022.848474 (PMC9121102; doi:10.3389/fpls.2022.848474)

T=Seita.6G192900.1_Q=Sit−miR166d_S=173

category=2_p=0.999804733205179

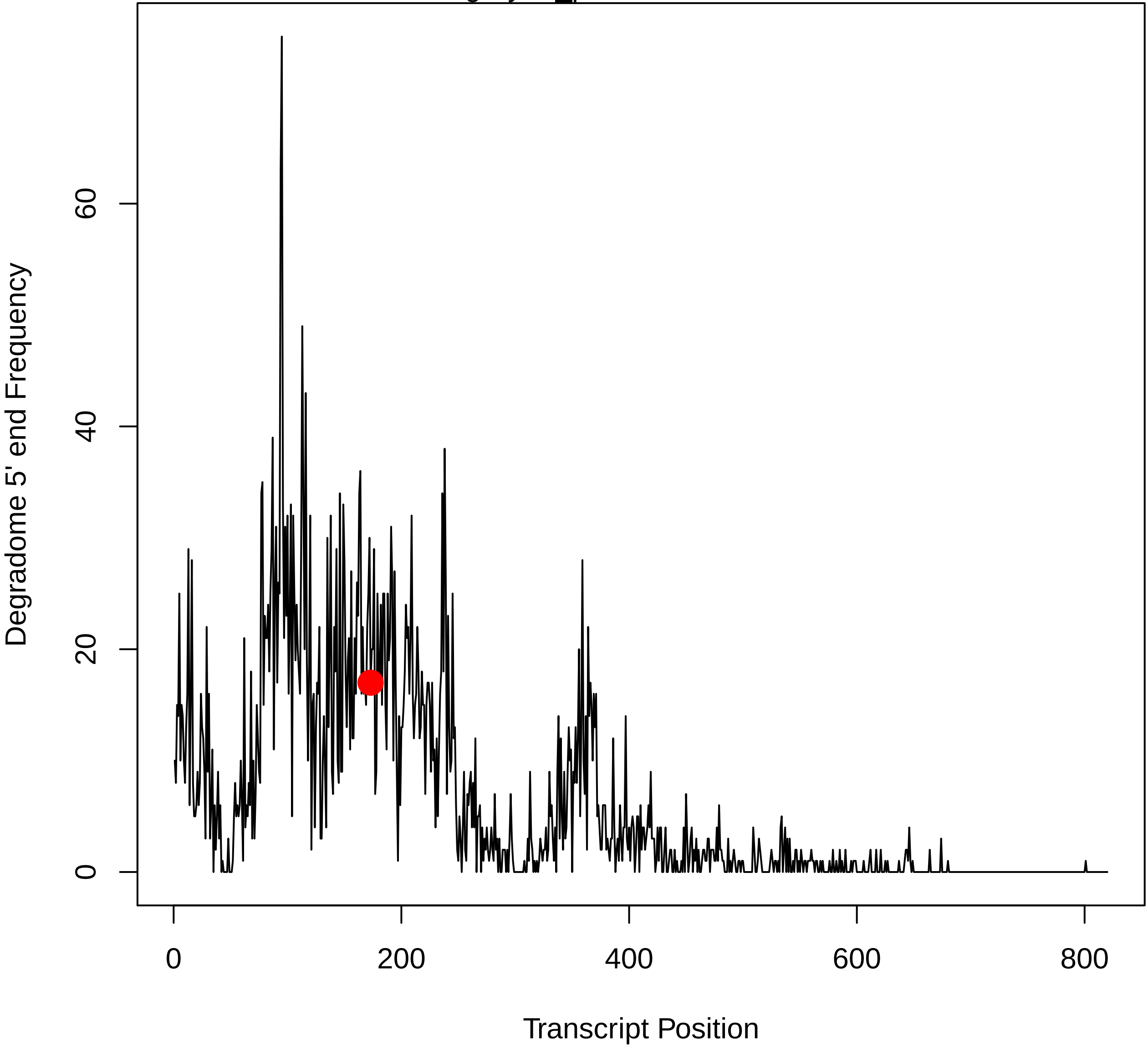

Supplement: Supplementary file 2 [file Data_Sheet_2.zip › Sit-miR166d_Seita.6G192900.1_173_TPlot.pdf]
